# Composition of PAHs in Biochar and Implications for Biochar Production

**DOI:** 10.1021/acssuschemeng.2c00952

**Published:** 2022-05-11

**Authors:** Wolfram Buss, Isabel Hilber, Margaret C. Graham, Ondřej Mašek

**Affiliations:** † Research School of Biology, Australian National University, 134 Linnaeus Way, 2601 Canberra, Australia; ‡ UK Biochar Research Centre, School of Geosciences, University of Edinburgh, Crew Building, Alexander Crum Brown Road, EH9 3FF Edinburgh, U.K.; § Methods Development and Analytics, Agroscope, Reckenholzstrasse 191, 8046 Zurich, Switzerland; ∥ School of Geosciences, University of Edinburgh, Crew Building, Alexander Crum Brown Road, EH9 3FF Edinburgh, U.K.

**Keywords:** biochar, pyrolysis, PAH, naphthalene, toxicity equivalent factor

## Abstract

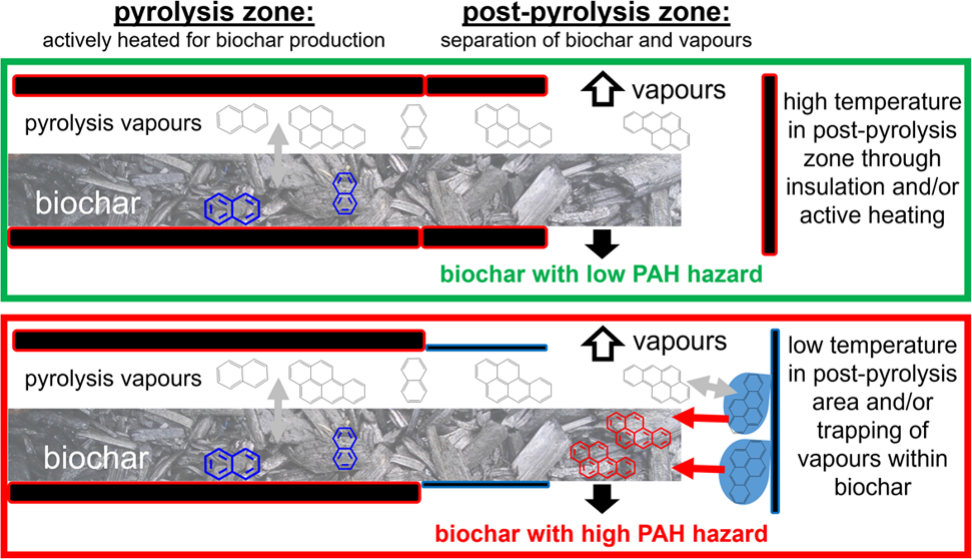

The content of polycyclic aromatic hydrocarbons (PAHs) in biochar has been studied extensively; however, the links between biomass feedstock, production process parameters, and the speciation of PAHs in biochar are understudied. Such an understanding is crucial, as the health effects of individual PAHs vary greatly. Naphthalene (NAP) is the least toxic of the 16 US EPA PAHs but comprises the highest proportion of PAHs in biochar. Therefore, we investigate which parameters favor high levels of non-NAP PAHs (∑16 US EPA PAHs without NAP) in a set of 73 biochars. On average, the content of non-NAP PAHs was 9 ± 29 mg kg^–1^ (median 0.9 mg kg^–1^). Importantly, during the production of the biochars with the highest non-NAP PAH contents, the conditions in the post-pyrolysis area, where pyrolysis vapors and biochar are separated, favored condensation and deposition of PAHs on biochar. Under these conditions, NAP condensed to a lower degree because of its high vapor pressure. In biochars not contaminated through this process, the average non-NAP content was only 2 ± 3 mg kg^–1^ (median 0.5 mg kg^–1^). Uneven heat distribution and vapor trapping during pyrolysis and cool zones in the post-pyrolysis area need to be avoided. This demonstrates that the most important factor yielding high contents of toxic PAHs in biochar was neither a specific pyrolysis parameter nor the feedstock but the pyrolysis unit design, which can be modified to produce clean and safe biochar.

## Abstract 2

Production of safe biochar, which is essential for environmental applications, can be ensured through adequate design of biochar production units.

## Introduction

Biochar is a solid, carbon-rich material produced from biomass at elevated temperatures (∼300 to 800 °C) in the absence of oxygen in a process called pyrolysis.^1^ When incorporated in soil, biochar can alter the soil’s physical, chemical, and biological properties, and studies show that, on average, biochar application increases plant growth.^2^ However, biochar can also contain contaminants that, when the biochar is applied to soil, can adversely affect soil flora and fauna.^3−7^

Various potentially toxic organic compounds, including polycyclic aromatic hydrocarbons (PAHs), are formed during biomass pyrolysis.^8−10^ PAHs are commonly defined as organic compounds composed only of C and H, which contain at least two condensed aromatic rings.^11^ Although PAHs commonly comprise complex mixtures of compounds^12^ in most cases, the sum of 16 US EPA PAHs are analyzed, published in the priority pollutant list of the US EPA proposed in the late 1970s, as those presenting the main toxicological and environmental concern in industrial wastewater.^13^ PAHs can have short-term, adverse effects on human and plant health, but it is their long-term carcinogenic, mutagenic, and teratogenic effects that are of particular concern.^12,14^ The chemical structures of the 16 priority PAHs vary greatly, and so do their properties and toxicities.

Among the 16 US EPA PAHs, naphthalene (NAP) is the only one with two aromatic rings and the least toxic one.^15^ NAP is not considered to be carcinogenic nor genotoxic, and the acute toxicity LD 50 (lethal dose to kill half of the population) for mice and rats is as high as 350–9500 mg kg^–1^ body weight.^16^ NAP is also the most volatile PAH (vapor pressure 0.087 mm Hg at 25 °C or Henry’s law constant (*H*_L_) of 45 Pa m^3^ mol^–1^) and evaporates significantly when present in soil; in a study, 30% of the NAP loss in soil was due to evaporation (benzo(a)pyrene (B(a)P) in comparison has a vapor pressure of 5.5 × 10^–9^ mm Hg at 25 °C or H_L_ of 0.046 Pa m^3^ mol^–1^).^17^ In the same study, the half-life of NAP was reported to be only 2 days, the shortest of 12 of the 16 US EPA PAHs tested.^17^ In some cases, NAP can be degraded in hours, e.g., in sediment that has previously been contaminated with PAHs and where microbial communities therefore adapted to the high PAH conditions.^12^

Considering the low hazard of NAP, the risk associated with biochars containing similar ∑16 US EPA PAHs content can vary greatly, depending on NAP content, which fluctuates significantly among different biochars, e.g., 14–63%^18^ and 11–83%.^19^ Many studies investigated the effect of pyrolysis on total PAH content in biochar, which demonstrates, for example, that the PAH content decreases with the carrier gas flow rate.^20−22^ Feedstock type and pyrolysis unit design also clearly influence PAH levels in biochar, with woody biochar typically showing lower PAH levels than grass biochars.^8,18,20,23−25^ Zhao et al. demonstrated that biomass enrichment with iron minerals prior to pyrolysis decreased PAH formation but increased the content of toxic PAHs in biochar.^26^ However, no clear correlation between PAH content and typical pyrolysis parameters, such as pyrolysis temperature or residence time in the heated zone, could be observed.^8,18,20,22,25,27^ In particular, there is still a lack of studies that take into account the different compositions of PAHs in large sets of biochars to give recommendations on safe biochar production.

In this study, the individual contents of the 16 US EPA PAHs were determined for a suite of 73 biochars produced from various feedstocks using different production conditions and in three well-monitored slow pyrolysis units of different operations and scales. The aim was to develop recommendations for the production of biochar with low PAH-related risk for human health and the environment. We therefore investigate whether high contents of non-NAP 16 US EPA PAHs in biochars can be linked to particular reactor design and pyrolysis conditions. To do so, the 10 biochars with the highest non-NAP PAH contents were studied thoroughly to pinpoint the reasons for the high PAH contents, based on a detailed and in-depth understanding of the production processes.

## Materials and Methods

### Pyrolysis Units

Three different pyrolysis units at the UK Biochar Research Center, University of Edinburgh, were used for biochar production.

(i) The “Stage I” pyrolysis unit is a small-scale, batch pyrolysis reactor with a vertical quartz tube (inner diameter 50 mm) that has a sample bed depth of around 200 mm and is heated up by a 12 kW infrared gold image furnace (P610C; ULVACRIKO, Yokohama, Japan). A condensation system was assembled to collect different fractions of condensable gases in cold traps, and noncondensable gases were collected in a gas bag for further analysis. A schematic of the unit with more details about the pyrolysis unit setup was published elsewhere.^28^
(ii) The “Stage II” pyrolysis unit is a continuous-screw pyrolysis unit (auger reactor) that uses an electrically heated split tube furnace with an inner diameter of 100 mm. After an initial nitrogen purge, the feed hopper transports the feedstock to a rotary valve, where it drops onto the furnace screw. The discharge chamber, where pyrolysis vapors and solids (biochar) are separated, was actively heated up with heating tapes (heating tape (HT) I and III), which were set to 500 and 400 °C, respectively. A third heating tape (HT II) heated the pipes that connect the discharge chamber with the afterburner, where the pyrolysis vapors are combusted using propane. At various parts of the pyrolysis units, pressure and temperature were measured. A schematic of the unit with more details about the unit can be found in Buss et al.^29^
(iii) The “Stage III” pyrolysis unit is a pilot-scale rotary kiln with a heat tube length of 2.8 m, an inner diameter of 244 mm, an angle of 0.5°, and a rotational speed of 1–7 rpm. A biomass hopper with a feed screw delivers the feedstock to the rotary kiln, where it is heated to a maximum temperature of 850 °C. The discharge chamber separates pyrolysis vapors from solids that drop on a cooling screw that transports the char to a nitrogen-purged discharge drum. The vapors are channeled into an afterburner, where they are combusted with propane and the exhaust gases are released. Temperature and pressure were monitored at different entry points within the heat tube. A schematic of the unit and more details are available in Buss et al.^30^

### Biochars

Seventy-three biochars were produced from 14 different feedstocks in the three different pyrolysis units described in the section above. The parameters controlled during the production included the highest treatment temperature (HTT), residence time at HTT, and carrier gas flow rates. Besides changes in the production conditions, changes to the pyrolysis unit setup were implemented. More details on biochar production can be found in the Supporting Information. In addition, four different feedstock pretreatments (K-doping, washing, drying, increase of moisture content) and one biochar post-treatment (heating at 200 °C for 20 h) were applied, which were part of previous studies. The biochars were chosen to relate PAH content and composition, and in particular, PAH toxicity, to feedstock properties (e.g., moisture or ash content) and biochar production conditions. All 73 biochars and their production conditions are displayed in Table 1, and more details, including PAH levels and references, where appropriate, can be found in Table S1.

**Table 1:** Production Conditions of 73 Biochars^a^

biochar ID	feedstock	unit	HTT (°C)	RT (min)	HR (°C min^–1^)	CGF (L min^–1^)
SWP II-350-10-0	softwood pellets II	stage I	350	10	5	0
SWP II-350-10-0.33	softwood pellets II	stage I	350	10	5	0.33
SWP II-350-10-0.66	softwood pellets II	stage I	350	10	5	0.67
SWP II-350-40-0	softwood pellets II	stage I	350	40	5	0
SWP II-350-40-0.33	softwood pellets II	stage I	350	40	5	0.33
SWP II-350-40-0.66	softwood pellets II	stage I	350	40	5	0.67
SWP II-650-10-0	softwood pellets II	stage I	650	10	5	0
SWP II-650-10-0.33	softwood pellets II	stage I	650	10	5	0.33
SWP II-650-10-0.66	softwood pellets II	stage I	650	10	5	0.67
SWP II-650-40-0	softwood pellets II	stage I	650	40	5	0
SWP II-650-40-0.33	softwood pellets II	stage I	650	40	5	0.33
SWP II-650-40-0.66	softwood pellets II	stage I	650	40	5	0.67
WSP II-350-10-0	straw pellets	stage I	350	10	5	0
WSP II-350-10-0.33	straw pellets	stage I	350	10	5	0.33
WSP II-350-10-0.66	straw pellets	stage I	350	10	5	0.67
WSP II-350-40-0	straw pellets	stage I	350	40	5	0
WSP II-350-40-0.33	straw pellets	stage I	350	40	5	0.33
WSP II-350-40-0.66	straw pellets	stage I	350	40	5	0.67
WSP II-650-10-0	straw pellets	stage I	650	10	5	0
WSP II-650-10-0.33	straw pellets	stage I	650	10	5	0.33
WSP II-650-10-0.66	straw pellets	stage I	650	10	5	0.67
WSP II-650-40-0	straw pellets	stage I	650	40	5	0
WSP II-650-40-0.33	straw pellets	stage I	650	40	5	0.33
WSP II-650-40-0.66	straw pellets	stage I	650	40	5	0.67
DNX-350	*Arundo donax*	stage II	350	20	n/a	1
DNX-450	*A. donax*	stage II	450	20	n/a	1
DNX-550	*A. donax*	stage II	550	20	n/a	1
DNX-650	*A. donax*	stage II	650	20	n/a	1
DNX-750	*A. donax*	stage II	750	20	n/a	1
DW-350	demolition wood	stage II	350	20	n/a	1
DW-450	demolition wood	stage II	450	20	n/a	1
DW-550	demolition wood	stage II	550	20	n/a	1
DW-650	demolition wood	stage II	650	20	n/a	1
DW-750	demolition wood	stage II	750	20	n/a	1
MC-350	miscanthus chips	stage II	350	20	n/a	1
MC-350-high ash	miscanthus chips	stage II	350	20	n/a	1
MC-350-low ash	miscanthus chips	stage II	350	20	n/a	1
MC-450	miscanthus chips	stage II	450	20	n/a	1
MC-450-dry	miscanthus chips	stage II	450	20	n/a	1
MC-450-wet	miscanthus chips	stage II	450	20	n/a	1
MC-550	miscanthus chips	stage II	550	20	n/a	1
MC-550-dry	miscanthus chips	stage II	550	20	n/a	1
MC-550-high ash	miscanthus chips	stage II	550	20	n/a	1
MC-550-low ash	miscanthus chips	stage II	550	20	n/a	1
MC-550-wet	miscanthus chips	stage II	550	20	n/a	1
MC-750	miscanthus chips	stage II	750	20	n/a	1
MC-750-dry	miscanthus chips	stage II	750	20	n/a	1
MC-750-high ash	miscanthus chips	stage II	750	20	n/a	1
MC-750-low ash	miscanthus chips	stage II	750	20	n/a	1
MC-750-wet	miscanthus chips	stage II	750	20	n/a	1
WC-350	willow chips	stage II	350	20	n/a	1
WC-550	willow chips	stage II	550	20	n/a	1
WC-750	willow chips	stage II	750	20	n/a	1
AD-550	sewage sludge AD	stage II	550	20	n/a	0
AD-700	sewage sludge AD	stage II	700	20	n/a	0
SS I-550	sewage sludge I	stage II	550	20	n/a	0
SS I-700-no HT I	sewage sludge I	stage II	700	20	n/a	0
FWD-550	food waste AD	stage II	550	20	n/a	1
WHI-550	water hyacinth	stage II	550	20	n/a	1
WSI-550	wheat straw	stage II	550	20	n/a	1
SWP I-550-no HT III	softwood pellets I	stage II	550	20	n/a	1
SWP I-550-purge 2 L min^–1^	softwood pellets I	stage II	550	20	n/a	1
SS II-350	sewage sludge II	stage III	350	20	n/a	10
SS II-450	sewage sludge II	stage III	450	20	n/a	10
SS II-550	sewage sludge II	stage III	550	20	n/a	10
SS II-650	sewage sludge II	stage III	650	20	n/a	10
SS II-750	sewage sludge II	stage III	750	20	n/a	10
SWP I-550-VC	softwood pellets I	stage III	550	20	n/a	10
SWP I-550-LC	softwood pellets I	stage III	550	20	n/a	10
SWP I-550-NC	softwood pellets I	stage III	550	20	n/a	10
SWP I-550-VC-200 T	softwood pellets I	stage III	550	20	n/a	10
SWP I-550-NC-200 T	softwood pellets I	stage III	550	20	n/a	10
SWP I-550-LC-200 T	softwood pellets I	stage III	550	20	n/a	10

a More explanations on pre-/post-treatments and unique factors during the particular pyrolysis runs can be found in Table S1. Biochars are abbreviated (biochar ID) in the following way: feedstock–HTT–further production conditions or pre-/post-treatments. HTT, highest treatment temperature; RT, residence time at HTT; HR, heating rate (batch process only); CGF, nitrogen carrier/inert gas flow rate; AD, anaerobic digestate; n/a, not available.

### PAH Extraction and Analysis

Every solid material with a different particle size is considered heterogeneous.^31^ Accurate and precise determination of PAHs in biochar largely depends on the homogeneity of the material or rather on the reduced heterogeneity. This is important for PAH analysis because the smaller the particle, the higher the PAH content.^32^ Furthermore, the relative standard deviation (RSD) of PAH levels is concentration-dependent^31^ in the way that the lower the concentration, the higher the RSD. Hence, the biochar needs to be thoroughly mixed prior to analysis (described below) to obtain a representative subsample. Under these circumstances, an RSD between 10 and 20% is considered good for PAHs in biochar.^31^

Representative samples were obtained by mixing the biochar samples manually, taking a subsample of around 1/10 of the total amount produced, grinding it with mortar and pestle, taking a homogenized subsample (2 g), and transferring it into a sample tub. Of this sample, ∼1 g was finally used for the extraction and PAH analysis.

Due to the strong sorption of organic substances by biochar, the method recommended for PAH extraction from biochar is a 36-h Soxhlet extraction using toluene.^33^ The analyses were conducted by Northumbrian Water Scientific Services (Newcastle, U.K.). As previously described,^20^ 100 mL toluene was added to 1 g of ground biochar that was subjected to Soxhlet extraction for 36 h, and the resulting extract was reduced to 1 mL and analyzed by GC-MS (Agilent 6890 GC plus 5975c MS). A six point calibration curve (1, 2, 5, 10, 20, and 50 mg L^–1^ from a stock solution of 2000 mg L^–1^) was run with all 16 US EPA PAHs (naphthalene, acenaphthylene, acenaphthene, fluorene, phenanthrene, anthracene, fluoranthene, pyrene, benzo(a)anthracene, chrysene, benzo(*b*)fluoranthene, benzo(*k*)fluoranthene, benzo(a)pyrene, (B(a)P), indeno(1,2,3-*cd*)pyrene, dibenz(*a,h*)anthracene, benzo(*ghi*)perylene). Five deuterated PAHs (NAP-d8, acenaphthene-d10, phenanthrene-d10, chrysene-d12, perylene-d12) compensated for the losses of the respective native compounds and were spiked into the solvent after extraction at 20 mg L^–1^. The correlation coefficient of all PAHs was >0.997. The limit of detection (LOD) was 0.10 mg kg^–1^. Extraction blanks (<3× LOD) were analyzed with each batch of samples and subtracted from all samples. The precision of extraction and GC-MS analysis was obtained by measurement of three replicates of some biochar samples and was <20%.

The individual PAH contents for all 73 biochars are reported in Table S2. Total PAH contents for 46 biochars^20^ and individual PAH contents for 6 biochars^9,34^ were already reported previously. Throughout the manuscript, the ∑16 US EPA PAH content without NAP is referred to as “non-NAP PAHs.”

## Results and Discussion

### PAH Composition in 73 Biochars

The content of ∑16 US EPA PAHs in all 73 biochars investigated in this study ranged from 1.2 to 232 mg kg^–1^ with an average of 27 ± 36 mg kg^–1^ (Table 2). The range of PAH contents is comparable to biochars in other studies, i.e., 0.07–355 mg kg^–1^.^8,18−20,33,35^

**Table 2:** Content of the Sum (∑) 16 US EPA PAHs, NAP, Proportion of NAP in Relation to ∑16 US EPA PAHs, Non-NAP PAH Content, Proportion of Non-NAP PAHs in Relation to ∑16 US EPA PAHs, and Toxicity Equivalent Quantity (TEQ) of All 73 Biochars, the 10 Biochars with the Highest Non-NAP PAH Content (Group 1), and the Remaining 63 Biochars (Group 2)^a^

	∑16 EPA PAHs (mg kg^–1^)			NAP content (mg kg^–1^)			NAP proportion (%)			non-NAP content (mg kg^–1^)			non-NAP proportion (%)			B(a)P-TEQ (mg kg^–1^)		
	mean	SD	median	mean	SD	median	mean	SD	median	mean	SD	median	mean	SD	median	mean	SD	median
all 73 biochars	27	36	20	17	16	14	83	22	90	9	29	0.9	17	22	10	1.4	4.1	0.05
group 1	85	65	53	27	14	26	39	20	36	58	61	35	61	20	64	8.3	8.4	4.4
group 2	18	16	14	16	15	13	89	12	90	2	3	0.5	11	12	8	0.34	0.9	0.04

a Values given as mean, standard deviation (SD), and median.

**Figure 1:**
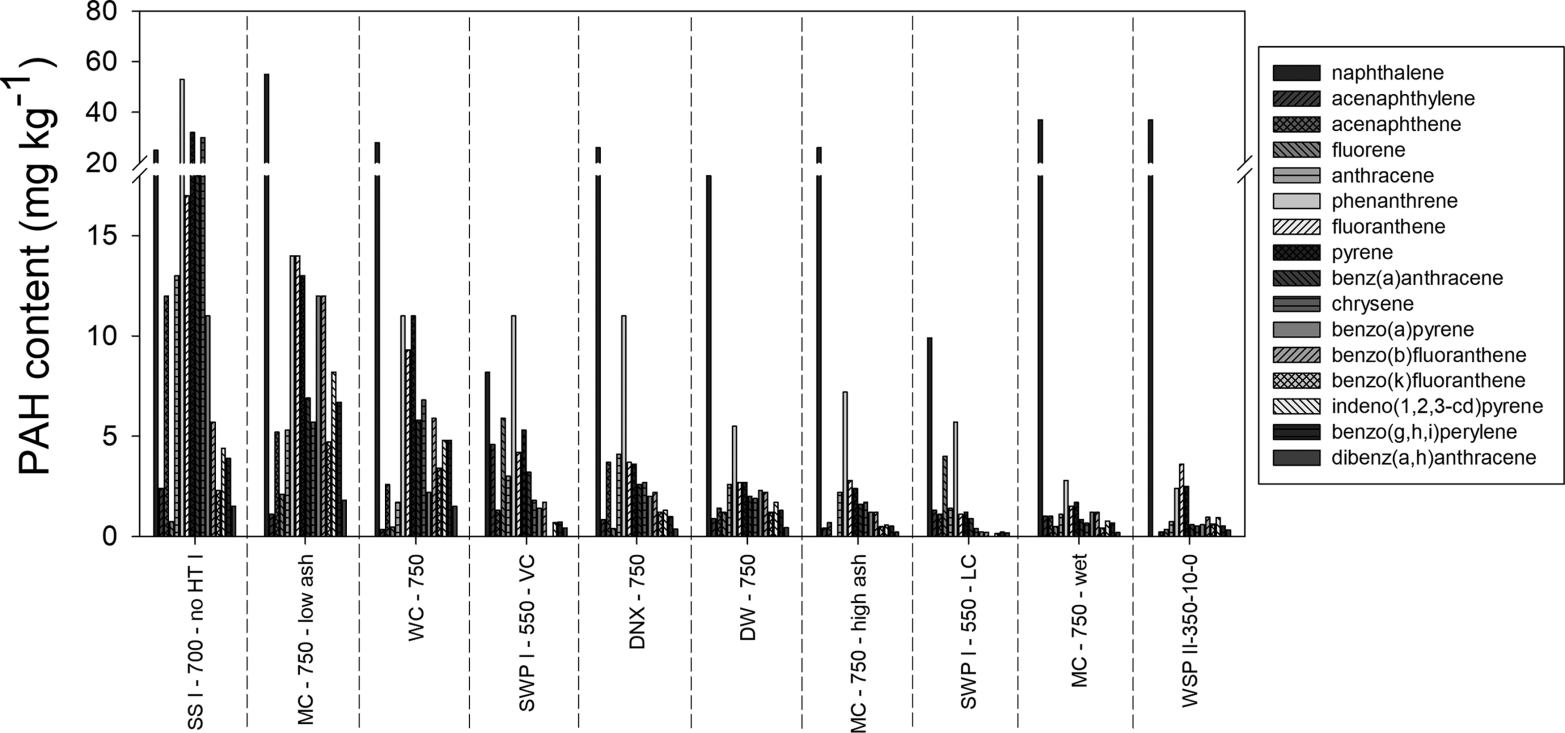
Individual concentration of 16 US EPA PAHs (mg kg^–1^) in the 10 biochars with the highest non-NAP PAH concentrations (group 1). Biochars are abbreviated in the following way: feedstock–HTT–further production conditions or pre-/post-treatments (Table S1).

Investigating the composition of PAHs in more depth proved to be challenging, as in many biochars, most individual PAH compounds were below the detection limit of 0.10 mg kg^–1^ (Table S2). Therefore, in Figure 1, the 10 biochars with the highest non-NAP PAH contents are displayed, where all of the individual PAHs have levels above the detection limit. The results showed that, except for the biochar with the highest PAH content (“SS I–700–no HT I”), NAP was the compound with the highest content of all individual PAHs (Figure 1). The PAH with the second-highest content in this set of 10 biochars was phenanthrene that was also the PAH with the second highest content, on average, in all 73 biochars in this study (mean 1.8 mg kg^–1^) and in biochars in other studies.^18,33,36^ The contents of the other 3-ring PAHs and more toxic 5- and 6-ring PAHs were typically slightly lower than the contents of 4-ring PAHs (Figure 1).

Taken all 73 biochar together, NAP was by far the most dominating PAH with 83 ± 22% of the total PAH contents (Table 2), which is in agreement with Fagernäs et al.^37^ and the 525 °C temperature biochars in Kloss et al.,^24^ where the proportion of NAP was also >80%. In a review, taking into account various biochars produced in rotary kilns, the NAP contents were between 30 and 80%.^38^ The range of values in our study (∼11 to 100%) was even broader, which in parts can be explained by the way the percentage of NAP of the sum of PAHs was calculated with individual PAH contents smaller than the limit of detection (LOD) (0.10 mg kg^–1^) not taken into account. If the LOD is used instead of a value of zero, the average proportion of NAP decreases to 72 ± 21%, which is well in the range reported for rotary kiln biochars.^38^ The reason for the high content of NAP in many biochars and why its content fluctuates so widely is unclear. To investigate this further, mechanistic studies are needed.

**Figure 2:**
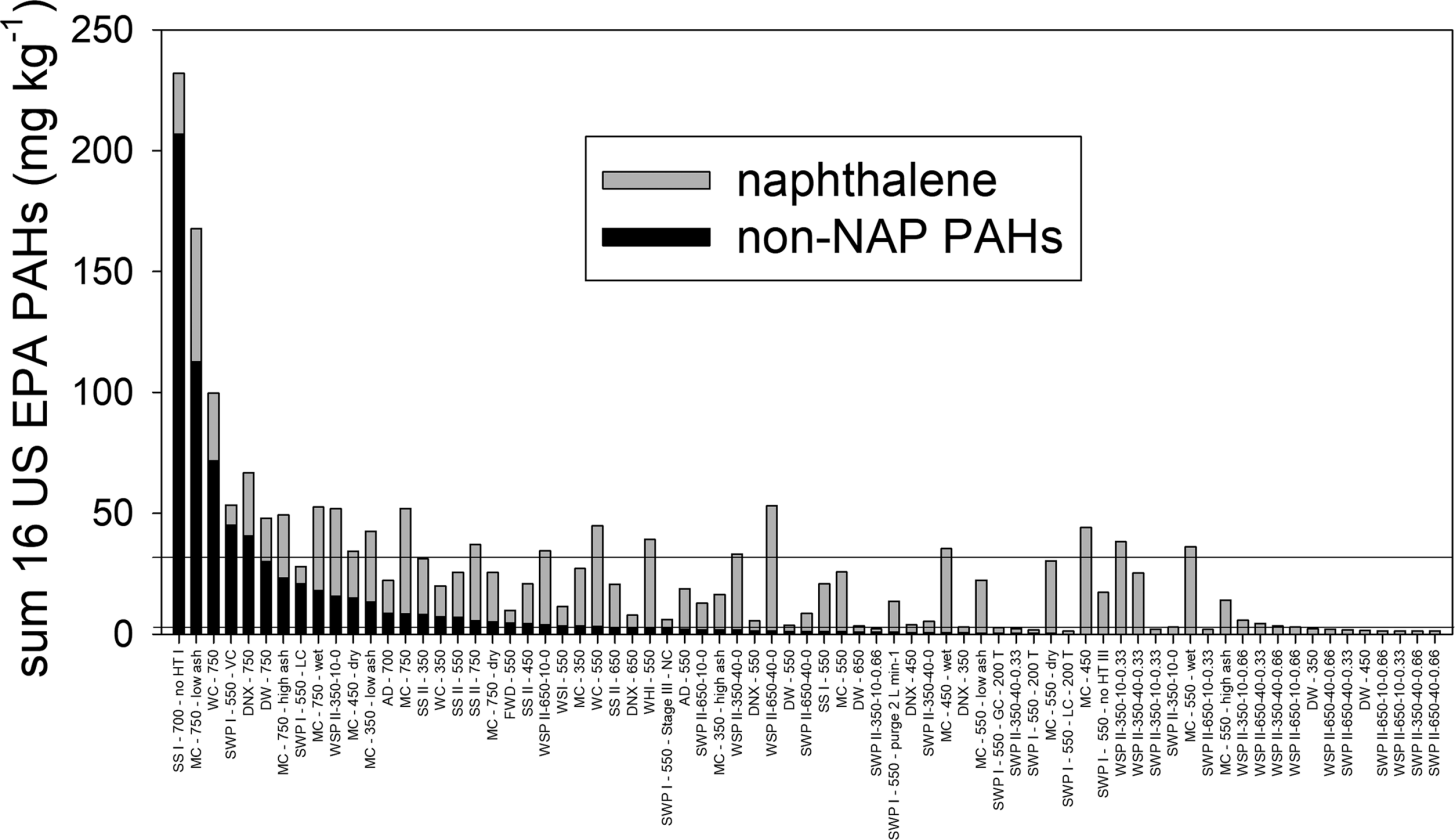
Content of the sum (∑) of the 16 US EPA PAHs in 73 biochars (mg kg^–1^) with proportions of naphthalene (NAP) and non-NAP PAHs. Biochars are abbreviated in the following way: feedstock–HTT–further production conditions or pre-/post-treatments (Table S1). PAH threshold values are indicated: the upper and lower lines are the EBC threshold values for class IV biochar (“EBC-Material”) of 30 mg kg^–1^ and class I (“EBC-Feed”) and II (“EBC-AgroBio”) biochar of 4 mg kg^–1^.

**Figure 3:**
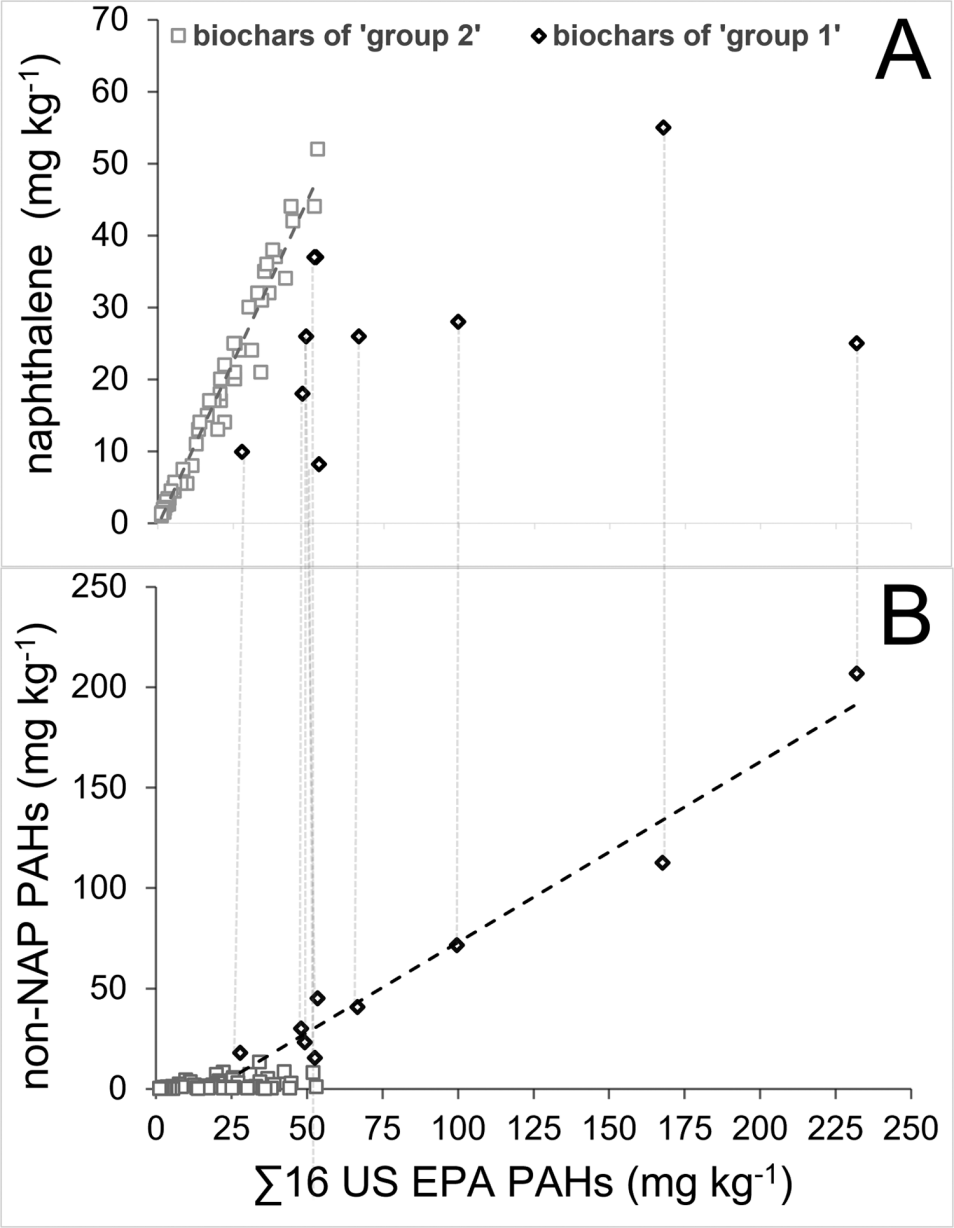
Contents of naphthalene (NAP) (A) and non-NAP PAHs (B) of 73 biochars vs the sum (∑) of the 16 US EPA PAHs (all in mg kg^–1^). The black squares show group 1, which are the 10 biochars with the highest non-NAP PAH concentrations, while the gray squares are the remaining biochars (group 2).

While in most of the biochars in our study, NAP comprised a very high proportion of the sum of 16 PAHs (>90%) (Figure 2), in some biochars, the non-NAP content dominated over NAP. In Figure 2, the biochars are arranged according to their non-NAP levels (largest to smallest values), which shows that the biochars with the highest non-NAP levels seem to have a lower proportion of NAP relative to their total PAH contents than those with a low non-NAP content (further on the right of the figure). Investigating this effect in more detail, we plotted the 16 US EPA PAHs in biochars vs their respective NAP and non-NAP contents and visually differentiated the 10 samples with the highest non-NAP levels (“group 1”) from the remaining samples (“group 2”) (Figure 3A,B). In group 2 (*n* = 63, low non-NAP content), there is a linear relationship of NAP with the total 16 US EPA PAHs (Figure 3A), while in group 1 (*n* = 10, high non-NAP content), there is a linear relationship of non-NAP levels with total 16 US EPA PAHs (Figure 3B). In biochar group 1, NAP represents only 40% of the PAHs measured; in group 2, it represents 90% (Table 2). There is a statistically significant difference between the NAP content in the two groups (Mann–Whitney *U* test; *p*-value <0.001). This suggests that the pathways of contamination for NAP and non-NAP PAHs are different.

To reveal the cause for this distribution of PAHs in these biochars and to present options for avoiding high non-NAP PAH biochar production, the production process of some of the biochars with the highest non-NAP contents are discussed in detail in the following section.

### Reasons for High Contents of Non-NAP PAHs in Biochar

When comparing the production conditions and feedstocks of the 10 biochars with the highest non-NAP PAH contents (Table S3), it becomes apparent that the high contents of PAHs cannot be associated with a particular feedstock type, which is in line with other studies, e.g., Bucheli et al.^38^ A large variety of feedstocks, from plant residues (softwood pellets, wheat straw pellets, miscanthus and willow), sewage sludge, and demolition wood yielded biochars with elevated levels of non-NAP PAHs under certain, but not all, production conditions. The increased non-NAP PAHs content could also not be unanimously ascribed to a particular pyrolysis temperature nor pyrolysis unit.

### Faults during Operation of the Stage III Pyrolysis Unit

Two of the biochars were produced during Stage III pyrolysis unit runs (pilot-scale rotary kiln), where irregularities led to the contact of biochar with pyrolysis vapors/liquids.^30^ The VC (vapor-contaminated) and LC (liquid contaminated) biochars, named due to their respective way of contamination during pyrolysis, were produced from softwood pellets at 550 °C with the Stage III pyrolysis unit (SWP I pyrolyzed at 550 °C, Table S1).^9,30^ During the “VC biochar”-production, a connecting pipe to the combustion chamber was restricted and eventually blocked due to a buildup of tars, resulting in backflow of pyrolysis vapors that contaminated the biochar (vapor-contaminated biochar). During the production of “LC biochar,” the discharge chamber walls (separation of pyrolysis vapors and biochar) were much cooler than the pyrolysis vapors due to insufficient insulation and, therefore, the vapors in the pyrolysis gas condensed on the walls, and the liquids contaminated the biochar (liquid contaminated biochar). This caused a considerable increase of PAH levels and in particular, elevated levels in non-NAP PAHs over a sample produced under the same production conditions and from the same feedstock but after the discharge chamber and the previously blocked tube were cleared and insulated (SWP I pyrolyzed at 550 °C, NC biochar, Table S1).^9^

### Biochars Produced at 750 °C Using the Stage II Unit

**Figure 4:**
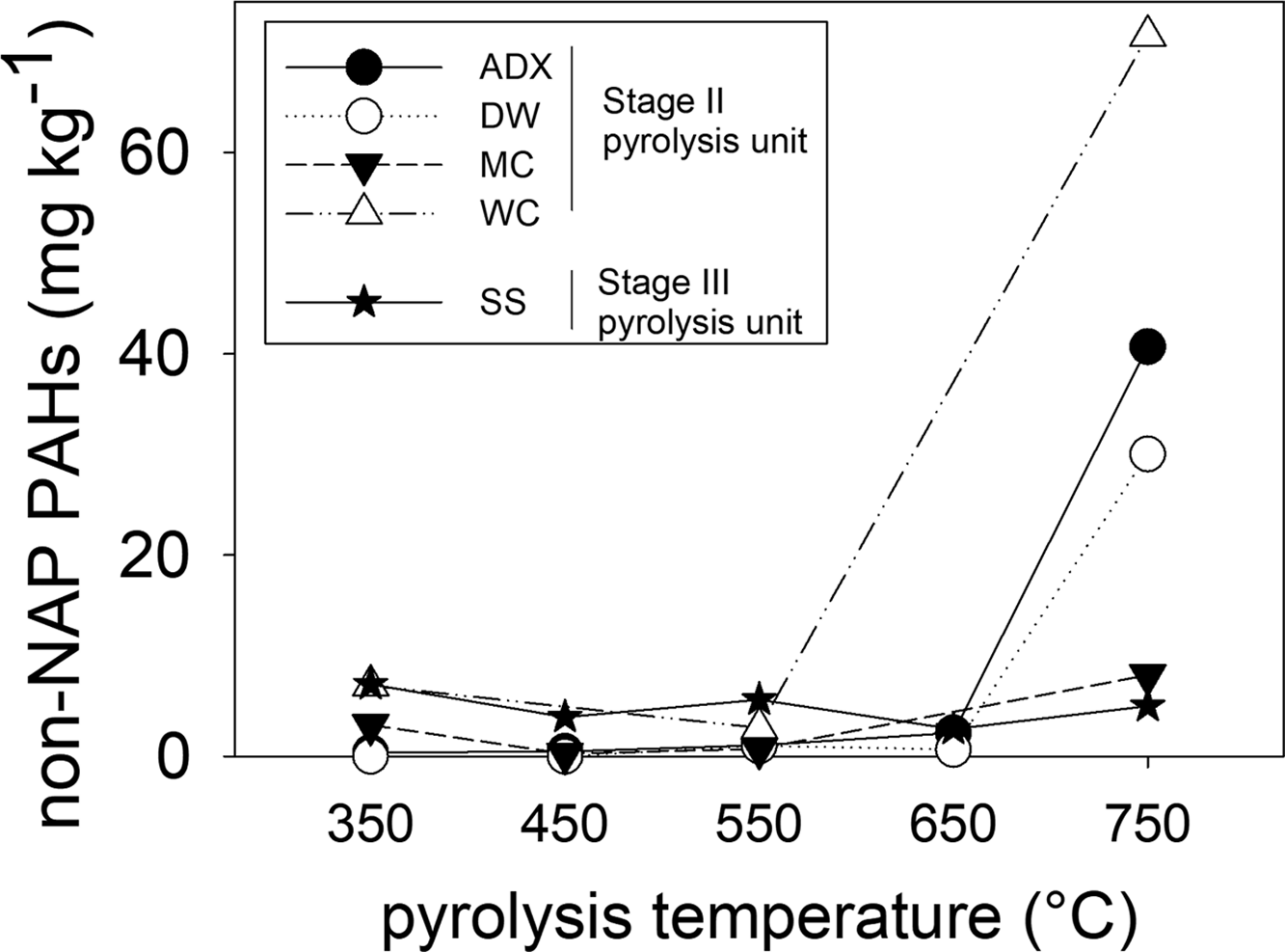
Effect of pyrolysis temperature on non-NAP PAH content (mg kg^–1^) in biochars from different feedstocks. The biochars were produced from four feedstocks in the Stage II pyrolysis unit (ADX, *A. donax*; DW, demolition wood; MC, miscanthus chips; WC, willow chips) and one feedstock in the Stage III pyrolysis unit (SS, sewage sludge).

Six of the 10 biochars with the highest non-NAP PAH content were produced at HTT of 750 °C in the Stage II pyrolysis unit (bench-scale auger pyrolysis unit). Analysis of total PAHs in these and other biochar samples, as reported in Buss et al.^20^ showed that the biochar produced in the Stage II unit at 750 °C had significantly higher total PAH contents than the biochar produced at lower temperatures in the same unit. This marked increase in PAH content with HTT increase to 750 °C becomes even more pronounced when only non-NAP PAHs are considered (Figure 4). Compared to the non-NAP PAH contents of the biochars produced at 350 °C, the biochars produced at 750 °C have ∼300-fold (DW, 30 mg kg^–1^ at 750 °C, <LOD at 350 °C), ∼100-fold (DNX), ∼2.6-fold (MC) and ∼10-fold (WC) higher contents. This trend is not supported for biochar produced in the other continuous pyrolysis unit (Stage III unit), where the non-NAP PAH content in biochar produced at 750 °C was lower (5.0 mg kg^–1^) than that in the biochar produced at 350 °C (7.2 mg kg^–1^) (Figure 4).

After detailed investigations, in Buss et al.,^20^ we identified the temperature of the heating tapes in the discharge chamber of the Stage II pyrolysis unit as the cause of the high PAH content in biochar. The discharge chamber is the area where pyrolysis vapors and solids are separated. The temperatures of the heating tapes (between 400 and 500 °C) were too low to prevent the condensation of pyrolysis vapors at the discharge chamber walls when the pyrolysis zone was operating at 750 °C. The condensed vapors subsequently deposited on biochar. Although PAH condensation and deposition will happen at all HTTs, this effect is only apparent in biochars produced at 750 °C because the PAH formation during pyrolysis (sum of PAHs recovered in all three pyrolysis products) is substantially higher at temperatures >700 °C^39−43^ and, therefore, pyrolysis vapors produced at 750 °C contain much more PAHs than vapors produced at lower HTT. Since the temperature in the discharge chamber of the Stage II unit is fixed at 400–500 °C irrespective of the HTT in the pyrolysis zone, there is a strong temperature gradient between the pyrolysis zone and discharge chamber when the pyrolysis zone operates at 750 °C and, therefore, PAH condensation in the discharge chamber is high (illustrated in Figure S1). In contrast, the discharge chamber of the Stage III unit is passively heated through insulation of the post-pyrolysis zone (after initial issues with post-pyrolysis contamination in the uninsulated discharge chamber; discussed in the previous section). Therefore, the higher the pyrolysis temperature in the Stage III unit, the higher the discharge chamber temperature, which lowers the temperature gradient and reduces vapor condensation.

### Heating Tape Failure in the Discharge Chamber of the Stage II Unit

The biochar produced from anaerobically digested sewage sludge at 700 °C (“AD–700”) had a total PAH content of ∼22 mg kg^–1^ with a proportion of NAP of 63% (Table S1). The levels were comparable to the total PAH content in biochar produced at 550 °C from the same feedstock (∼19 mg kg^–1^, 90% NAP, Table S1) and the undigested sewage sludge biochar (SS I, ∼21 mg kg^–1^, 96% NAP, Table S1). However, SS I pyrolyzed at 700 °C had a total PAH content of ∼232 mg kg^–1^ (11% NAP, Table S1), of which non-NAP PAHs comprised ∼207 mg kg^–1^. After investigations, we noticed that during the production of this biochar sample, one of the heating tapes in the discharge chamber of the Stage II pyrolysis unit was faulty (HT I) and consequently, the temperature measured by a thermocouple located between the inner and outer wall of the discharge chamber was much lower (113 °C) than in comparable pyrolysis runs (e.g., “AD–700,” 198 °C). The temperature does not reflect the internal temperature within the discharge chamber, but it does indicate the relative difference between the two pyrolysis runs. As a result of the considerably lower temperature in the discharge chamber, it is very likely that PAHs condensed and deposited on biochar. This example illustrates that PAH contamination through condensation and deposition might be a common phenomenon in biochar production and can occur even due to small modifications or faults during the pyrolysis unit setup or its operating parameters.

### Post-Pyrolysis PAH Contamination

As the above examples illustrate, the biochars with high contents of non-NAP PAHs were most likely contaminated with pyrolysis vapors in the post-pyrolysis zone. The pyrolysis process itself is very effective in separating PAHs from pyrolysis solids; <1% of the PAHs synthesized during pyrolysis are typically found in biochar, and the remaining proportion resides in the pyrolysis liquids and gases.^37,39^ Under high temperatures in the pyrolysis zone, most PAHs are either evaporated or react with the biochar in the so-called secondary char formation process that is responsible for a significant part of the biochar production.^44,45^ However, when pyrolysis vapors condense in the post-pyrolysis stage, in areas much cooler than the furnace and PAHs deposit on biochar, they are not converted into but instead physically sorb onto biochar and thus contaminate the material. This still does not explain why the proportion of NAP is smaller in these biochars.

The processes determining the content and composition of PAHs in biochar within the furnace area (formation and evaporation) are complex and depend on a number of parameters.^20,27^ At lower temperatures (<∼300 °C), such as those that can occur in cool zones in the post-pyrolysis stage (discharge chamber), less formation and transformation reactions of organic compounds occur than at high temperatures in the pyrolysis zone; instead, condensation and deposition of PAHs present in the pyrolysis gas and vapors on biochar dominate. This depends on (i) the content of the PAH in the gas phase at the respective pyrolysis temperature; (ii) the (equilibrium) vapor pressure of the PAH (boiling point); and (iii) the temperature difference between the gases in the reaction chamber and the surface for potential condensation (discharge chamber wall or biochar). NAP has a much higher vapor pressure than higher molecular weight (HMW) PAHs, such as B(a)P (values in the introduction) and hence is more volatile and will remain in the gas phase to a higher degree (illustrated in Figure S1). This means that, of the B(a)P and NAP present in the gas phase, a larger proportion of B(a)P will condense and deposit as a liquid when the temperature in the discharge chamber falls. Therefore, in biochars contaminated via vapor condensation, as observed, it is expected that the proportion of HMW PAHs will increase relative to the amount of NAP.

### Recommendations for Biochar Production and Future Studies

Here, we demonstrate that the high levels of non-NAP PAHs were mainly the result of condensation and deposition of PAH in cool zones of the post-pyrolysis area rather than the result of the pyrolysis process itself. Therefore, the pyrolysis unit needs to be carefully designed to avoid cool zones along the whole biochar production path. A suitable design of biochar discharge chamber arrangements is crucial. Where the pyrolysis gases and solids travel concurrently through the pyrolysis unit, the discharge chamber, which separates pyrolysis solids and vapors, needs to be maintained at temperatures as close as possible to the HTT. Another option is to separate pyrolysis vapors from solids already within the pyrolysis reactor, e.g., using a counter-current arrangement, where pyrolysis gases are extracted close to the feedstock entry point, i.e., on the opposite end from the biochar discharge. Such a counter-current arrangement would not only have an impact on the quality of the biochar—yielding biochar with lower PAHs contents—but also could even increase the yield of biochar due to increased secondary char formation.^45^ Overall, this work emphasizes the importance of monitoring and controlling the pyrolysis process beyond just simple parameters such as HTT in the pyrolysis reactor to achieve production of good quality biochar.

In the literature, effects of various pyrolysis parameters on PAH contents in biochar were reported; in particular, numerous different effects of HTT on PAHs in biochar have been observed.^4,8,19,25,27,39,46^ Despite significant efforts, these studies have not provided a general relationship between biochar PAH contents and HTT. Based on our investigations, we suggest this is the case since important aspects of biochar production have not been sufficiently addressed by these studies, to allow development of such general understanding. In this study, we demonstrated that weaknesses in the design or in the operation of biochar production units can have a striking effect, resulting in high contents of non-NAP PAHs in biochar and which also significantly increases the total PAH content. This condensation effect of pyrolysis vapors in cooler zones during pyrolysis operations is of great significance and indeed surpassed the effects of HTT, carrier gas flow, or feedstock.

Our observation is supported by various studies that report the highest levels of PAHs in biochars produced under “uncontrolled field conditions”^8^ or in “traditional kiln or soil mounds,”^27,47^ where heat distribution is uneven and vapor release can be inhibited and cause condensation of PAHs on biochar. In De la Rosa et al., the highest levels of total (and toxic) PAHs were found in biochars produced at lower temperatures (400 °C) in a muffle furnace setup that did not allow for pyrolysis liquid release (nor sufficient PAH incorporation into the biochar polyaromatic matrix due to the low temperatures).^23^ The second-highest level of PAHs in De la Rosa et al. were found in biochars from traditional kilns, while biochar from a high-tech, continuous pyrolysis reactor demonstrated low levels of PAHs.^23^ It is important to note that PAH contents in biochars made from simple “Kon-Tiki” flame curtain pyrolysis—where volatiles can freely escape and hence are unlikely to condense—were also low.^48^ This demonstrates that safe biochar can be produced in the field with the right technology.

We recommend that future investigations of the relationships between biochar PAH contents closely monitor the temperatures in the different zones of the pyrolysis unit, in particular, the discharge chamber. This ensures that the biochars are compared on the same basis so that certain processes, such as deposition of PAHs, are not misinterpreted as effects of, for example, pyrolysis temperature or feedstock.

### PAH Composition in Biochar and Threshold Values

For a risk-based assessment of biochars, changes in the content of NAP are of little relevance due to NAP’s low carcinogenicity and toxicity and rapid degradation in soil.^12,16,49^ However, since both environmental legislation and biochar guidelines values are typically based on the sum of the 16 US EPA PAHs, the content of NAP often decides about compliance/noncompliance with PAH threshold values.

In Figure 2 and Table 3, two biochar guideline values for PAHs defined in the European Biochar Certificate (EBC) are shown; the lowest (4 mg kg^–1^, EBC-FEED or EBC-AgroBio) and the highest (30 mg kg^–1^, EBC-Material) (the International Biochar Initiative (IBI) values for biochar soil use are 6 mg kg^–1^ and 300 mg kg^–1^).^50,51^ When NAP is considered, 24 (33%) and 51 (70%) of the set of 73 biochars exceeded the EBC upper and lower guideline values, respectively. However, when NAP is excluded, only six biochars (8%) exceeded the 30 mg kg^–1^ threshold and 20 biochars (27%) the 4 mg kg^–1^ threshold, respectively. This shows the disadvantage of using the sum of 16 US EPA PAHs for evaluating the potential risk of PAHs in biochar, where all PAHs are weighted equally. Consequently, alternative ways of evaluating the risk of PAHs in biochar should be used.

**Table 3:** Number and Proportion of Biochars Out of the Set of 73 Exceeding Guideline Values^a^

		EBC class I and II, 4 mg kg^–1^	EBC class IV, 30 mg kg^–1^
∑16 EPA PAHs	number of biochars	51	24
	proportion	70%	33%
non-NAP PAHs	number of biochars	20	6
	proportion	27%	8%

a The total contents of the sum (∑) of the 16 US EPA PAHs (including NAP) and non-NAP PAHs were considered separately. The threshold values are taken from the European Biochar Certificate (EBC) (EBC class I, EBC-FEED; EBC class II, EBC-AgroBio; EBC class IV, EBC-Material).

Benzo(a)pyrene is the most investigated PAH and is often used as a reference point to compare the toxicities of all 16 US EPA PAHs.^15^ Its average content in all 73 biochars was 0.59 ± 1.82 mg kg^–1^, but most biochars showed contents below the limit of detection of 0.10 mg kg^–1^ (Table S2). The B(a)P contents correlate well with the non-NAP PAH contents in the higher and lower end of non-NAP PAH levels in this study (Figure S2; *R*^2^ = 0.84) and, consequently, could be used as an indicator for non-NAP PAHs and PAH-associated risk as already established for food products in the EU.^52^ The EBC has a threshold value for B(a)P in place for animal feed additive (0.025 B(a)P mg kg^–1^), yet in addition, it has a threshold value for ∑16 US EPA PAHs that needs to be met.

As an alternative to threshold values based on the sum of the US EPA 16 PAHs, Nisbet and LaGoy^49^ used B(a)P as a reference to set up toxicity equivalent factors (TEFs), normally of 1, based on the carcinogenicity of B(a)P. For comparison, NAP has a TEF of 0.001.^49^ Multiplying the TEFs with their respective PAH contents in biochar and summing up the values for all PAHs results in the toxicity equivalent quantity (TEQ). The mean TEQ based on the 16 US EPA PAHs for all 73 biochars in this study was 1.4 mg kg^–1^ B(a)P-TEQ (Table 2) and varied from 0 to 25 mg kg^–1^ B(a)P-TEQ. The TEQs are similar to the values in Wang et al.,^53^ who report an increase in TEQ with pyrolysis temperature. In our biochars produced in the temperature range 350–750 °C, we cannot confirm a temperature effect, except for higher TEQs in the 750 °C biochars produced in the Stage II pyrolysis unit (Figure S3), where contamination of biochars with vapors occurred. In Wang et al.^,^^53^ the number of outliers with higher TEQ increases in the biochars produced at higher pyrolysis temperature. It is likely that these biochars were contaminated through condensation and deposition of non-NAP PAHs in the post-pyrolysis area as our 750 °C biochars from the Stage II unit. When the outliers are included and the mean is calculated, this shows a misleading trend.

In the IBI biochar guidelines (update 2015),^51^ a threshold value based on the TEF approach was included, though only taking into account the eight most toxic PAHs (3 mg kg^–1^ B(a)P-TEQ). This value is only exceeded by the six most contaminated biochar of the total of 73 biochars analyzed in our study and shows that guide values based on the TEF method already exist but are, however, not legally binding.

To bring our TEQ values for all 16 US EPA in context, we compared them to urban and rural soil in the U.K. as published in Creaser et al.^54^ The mean TEQ in group 1 (8.3 B(a)P-TEQ kg^–1^) (Table 2) was higher than the value for U.K. urban soils of 1.74 B(a)P-TEQ kg^–1^, yet the mean of group 2 (0.34 B(a)P-TEQ kg^–1^) was lower than the mean TEQ of PAHs in rural soils (0.44 B(a)P-TEQ kg^–1^). Importantly, the median of the entire set of biochars in our study was only 0.05 mg kg^–1^, highlighting that the vast majority of biochars are safe for application to soil.

The 16 US EPA PAHs were set up for wastewater originally, where other PAHs rather than NAP tend to dominate.^55^ Overall, it does not seem advisable to use threshold values based on the sum of the content of 16 US EPA PAHs as they do not discriminate between the toxicity of the compounds, for instance, such as NAP and highly carcinogenic compounds, such as B(a)P, for humans and the environment. Treating each PAH equally does not seem fit for purpose. For biochar, this is a particular issue as NAP is the dominant compound and its content fluctuates widely, and hence total PAH contents do not appropriately reflect the toxicity and, together with exposure, the risk associated with biochars. Instead, the content of B(a)P or the TEF approach should be used. Alternatively, different threshold values could be established individually for NAP and the sum of the remaining 15 US EPA PAHs as done in the German Federal Soil Protection Ordinance for the soil-groundwater interface.^56^ In the German Federal Soil Protection Ordinance, the threshold value for NAP is 10 times higher than the value for the sum of 16 US EPA PAHs without NAP. Using such an approach, the lower EBC threshold value for PAHs in biochar, for example, could remain at 4 mg kg^–1^ but re-defined as 16 US EPA PAHs without NAP, and the NAP only threshold value could be set at 40 mg kg^–1^. This methodology would better reflect the hazards associated with PAHs in biochar.

To assess the bioavailability or bioaccessibility of PAHs in biochar represents yet another approach.^8,57^ All methods seem to be suitable for biochar; however, since the TEF approach is widely used in practice for assessing the risk of dioxins, we suggest that it should also be the method of choice for setting up future guidelines and legislation thresholds for PAHs in biochar.

## Conclusions

The analysis of PAHs in 73 biochars, together with a detailed investigation of the production conditions, showed that post-pyrolysis contact of pyrolysis vapors with biochar was the most important factor determining the content of non-NAP PAHs in biochar in this study. The NAP content was only marginally, if at all, influenced by this process, which can be explained by NAP’s high vapor pressure and therefore, low chance of condensation in cool zones of the pyrolysis unit. Post-pyrolysis contamination by condensation and deposition of PAHs was much more important than the effects of HTT, carrier gas flow rate, or feedstock choice in determining the non-NAP PAH levels in biochar. These findings are novel and of great significance for biochar research, applications, and relevant regulations.

To ensure the production of biochar with low non-NAP content and, thus, low PAH-related hazard, the pyrolysis unit design and operation mode must be modified to avoid conditions suitable for deposition of PAHs on biochar. We found the most common issue in our continuous production units (auger and rotary kiln) was insufficiently high temperature in the area where pyrolysis vapors and biochar are separated. This discharge chamber either needs to be actively heated and/or well insulated so that the furnace passively heats the area. In both cases, the temperature in the discharge chamber should be close to the temperature in the heated zone to minimize vapor condensation. The discharge chamber set up in a moving bed pyrolysis unit would be comparable to our auger and rotary kiln units, and therefore, although we did not test biochars from such units, our conclusions still hold true for other continuous pyrolysis units. Condensation and trapping of pyrolysis vapors on/within biochar in batch units, for example, in traditional kilns or muffle furnaces, caused by uneven heat distribution and because vapors cannot escape freely, also need to be avoided. Our study highlights that biochar with low total and non-NAP PAH contents can, in principle, be produced from different feedstock, on different scales, and using different technologies.

Lastly, with biochar PAHs being dominated by low-toxicity NAP, the use of threshold values in biochar standards and environmental regulations based on the sum of 16 US EPA PAHs that was introduced with wastewater in mind is not fit for purpose. New standards and regulations should set PAH limits based on their toxicity and perhaps even availability, e.g., using the TEF approach to appropriately reflect the hazards of different PAHs.

## Supporting Information Available

More details about biochar production; additional data in figure and table format; and a schematic illustration of post-pyrolysis zone PAH contamination of biochar (PDF)
Total and individual levels of PAHs for each biochar investigated in this study (XLSX)

## Abbreviations

PAH: polycyclic aromatic hydrocarbon
NAP: naphthalene
HTT: highest treatment temperature
HMW: high molecular weight
TEF: toxicity equivalent factor
non-NAP PAHs: 16 US EPA PAHs excluding naphthalene
