# Supplementary material for: Composition of PAHs in Biochar and Implications for Biochar Production
Source: ACS Sustain Chem Eng. 2022 May 11;10(20):6755–65. doi: 10.1021/acssuschemeng.2c00952 (PMC9131514; doi:10.1021/acssuschemeng.2c00952)
Supplement: Supplementary file 1 — sc2c00952_si_001.pdf [file sc2c00952_si_001.pdf]

Composition of PAHs in biochar and implications for biochar production

Wolfram Buss[a,b]*

Isabel Hilber[c]

Margaret C. Graham[d]

Ondřej Mašek[b]

[a] Research School of Biology, Australian National University, 134 Linnaeus Way, 2601 Canberra, Australia

[b] UK Biochar Research Centre, School of GeoSciences, University of Edinburgh, Crew Building, Alexander Crum Brown Road, EH9 3FF Edinburgh, U.K.

[c] Agroscope, Methods Development and Analytics, Reckenholzstr. 191, 8046 Zurich, Switzerland

[d] School of Geosciences, University of Edinburgh, Crew Building, Alexander Crum Brown Road, Edinburgh, EH9 3FF, UK

*Correspondence: wolfram.buss@anu.edu.au

Number of pages: 7

Number of figures: 3

Number of tables: 1

Two additional tables are supplied in excel format

Materials and Methods – details of biochar production

Of the 73 biochars, the production of 55 biochars was described previously which includes (respective references are also indicated in SI Table 1):

- 13 of the 19 marginal biomass-derived biochars described in Buss et al. (2016b)
- all 46 biochars in Buss et al. (Buss et al., 2016a) with 10 of the biochars are already being part of the 13 biochars in Buss et al. (2016b)
- and the six (post-treated) biochars described in Buss et al., 2015, Buss and Mašek (2016) and Buss and Mašek (2014).

In the following paragraphs, the production of the remaining 18 biochars is described:

Two biochars were produced from softwood pellets (SWP I) at 550°C by the Stage II pyrolysis unit but with following modifications to the unit's discharge chamber: (i) under normal circumstances the discharge chamber, where pyrolysis vapours and solids are separated, was heated up with two heating tapes (HT I at 500 °C and III at 400 °C) and the hot air from the nitrogen streams through the furnace at a rate of 4 L min^{-1} (Buss et al., 2016b). Here, however, the purge gas flow rate was turned down to 2 L min^{-1}, producing 'SWP I - 550 - purge 2 L min^{-1}'. (ii) 'SWP I - 550 - no HT III' was produced under identical conditions with the exception that, instead of turning down the nitrogen gas flow, HT III was not in use.

Four biochars were produced from sewage sludge (SS I) and anaerobically digested sewage sludge (AD) from Wessex water (Avonmouth, UK) that were both pyrolysed at a HTT of 550 and 700°C in the Stage II pyrolysis unit. During the production of SS I at 700°C the HT I in the discharge chamber was not in use.

Nine biochars were produced, to test the effect of moisture content on PAH content, six of them were already described in Buss et al. (2016a) (untreated samples). Miscanthus chips (MC) were pre-treated to give a range of moisture contents and were subsequently pyrolysed at 450, 550 and 750°C in the Stage II pyrolysis unit. To obtain dry miscanthus chips, the feedstock was oven-dried at 105°C until constant weight. To obtain high-moisture feedstock, tap water was sprayed onto the material. The samples were stored under ambient conditions. On the day the samples were pyrolysed, the moisture contents were: untreated sample 13.4 wt%, dried sample 0.28 wt% and wet sample 23.5 wt% (determined by drying as above).

Nine biochars were produced from miscanthus, to test the effect of ash content on PAH content, six of them were already described in Buss et al. (2016a) (untreated samples). To obtain low-ash biomass, the chips were washed twice in hot tap water and once with cold deionised water over night. To prepare high-ash biomass, K was added in the form of an aqueous solution of potassium acetate, which was evenly sprayed onto the dried miscanthus chips and restored the original moisture content (13.4%). The ash contents on dry weight basis determined by proximate analysis as described in Buss et al. (Buss and Mašek, 2014) (ashing at 900°C for 20 min in air) were: 4.2% for the untreated miscanthus, 3.2% for the washed sample and 7.4% for the potassium-spiked sample. The untreated and two treated samples (all with same moisture content at start of pyrolysis) were pyrolysed at 350, 550 and 750°C with the Stage II pyrolysis unit.

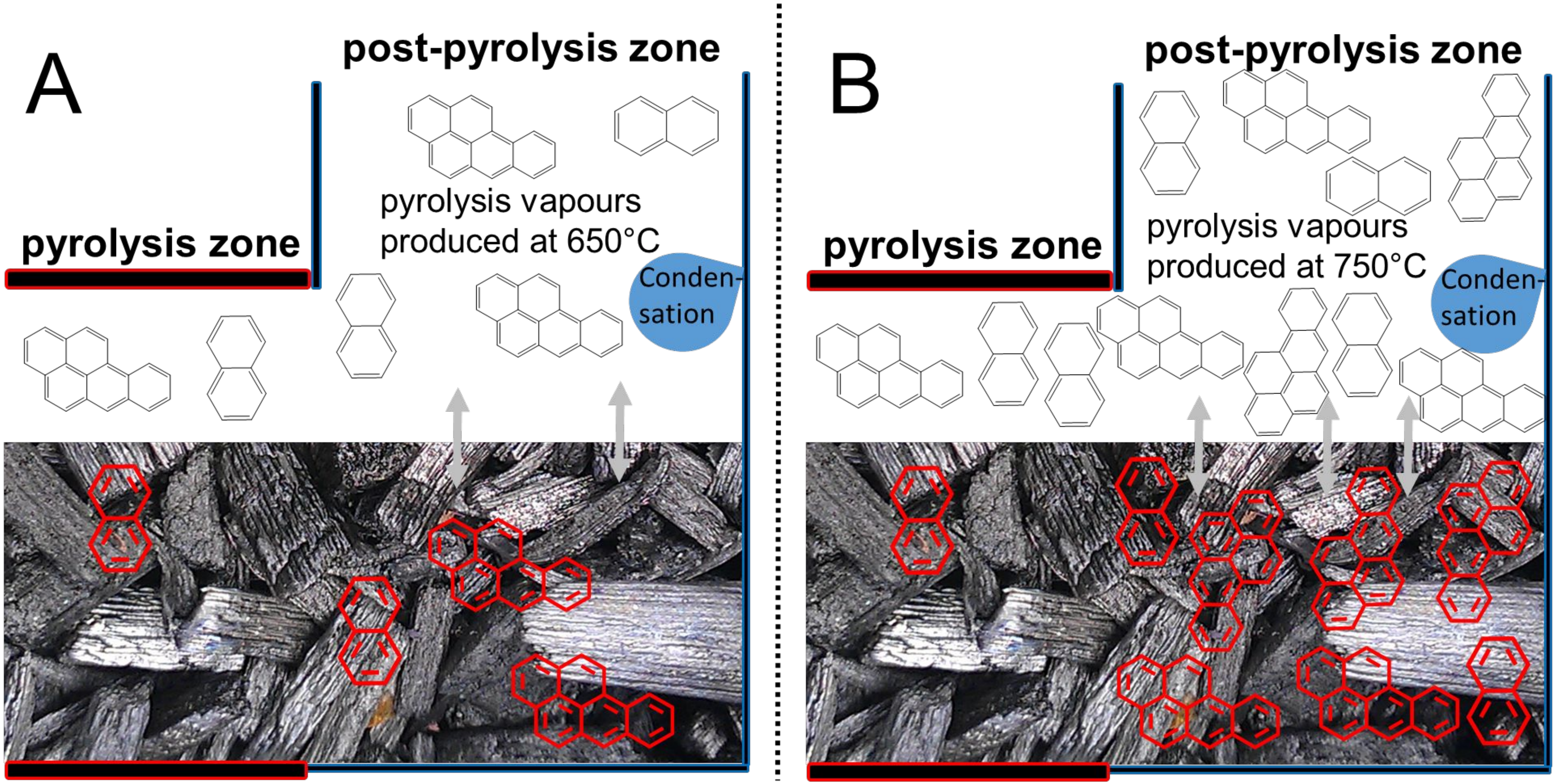

Figure S1: Schematic illustration of contamination of biochars by condensation of high-vapour pressure (here reflected by benzo(a)pyrene) and low-vapour pressure PAHs (here reflected by naphthalene) in the discharge chamber (post-pyrolysis area) of a pyrolysis unit with fixed temperature. In this particular pyrolysis unit, the temperature in the discharge chamber is set through the use of heating tapes that are fixed at the same temperature regardless of the temperature in the pyrolysis zone. PAHs with a higher vapour pressure condense to a higher extent than PAHs with a lower vapour pressure. The content of PAHs in pyrolysis vapours produced at 750°C (B) is much higher than the concentration of PAHs in pyrolysis vapours produced at lower temperatures, here reflected by 650°C (A). The ratio of PAHs in the gas phase *vs*. PAHs condensed on biochar is the same in (A) and (B) since the temperature in the discharge chamber area is the same. However, there is a higher level of PAHs condensing in (B), because there is a higher content of PAHs in pyrolysis vapours produced at 750°C.

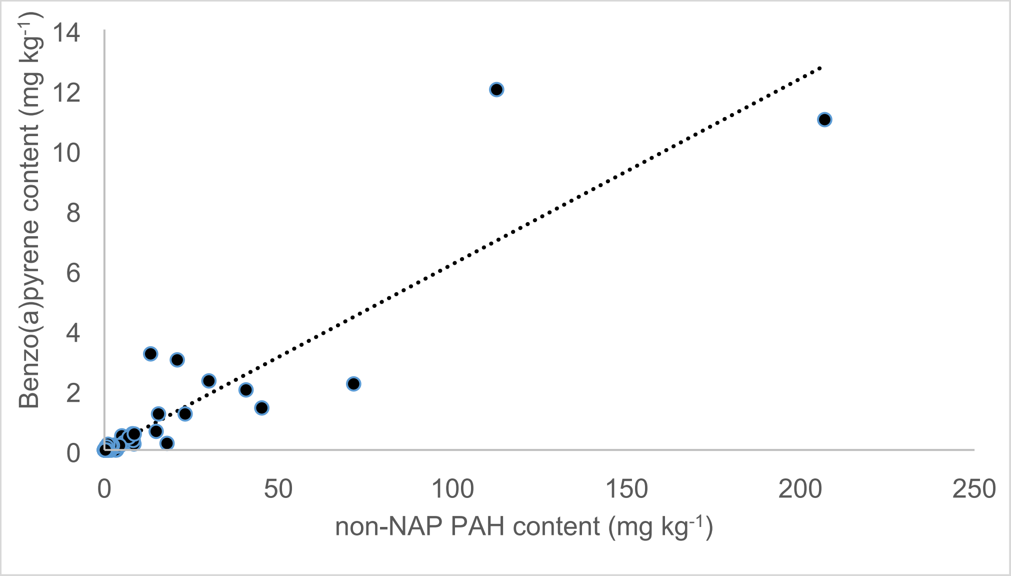

Figure S2: Correlation of benzo(a)pyrene content and non-NAP 16 US EPA PAH content in 73 biochars.

Table S1 and Table S2 are supplied in form of excel files.

Table S3: The ten biochars with the highest non-NAP PAH content and the feature most likely responsible for the high levels.

biochar	non-NAP mg kg $^{-1}$	PAH total mg kg $^{-1}$	NAP mg kg $^{-1}$	NAP %	feature most likely responsible for high non-NAP PAHs
SS I - 700 - no HT I	207	232	25	10.8	Stage II unit - heating tape in discharge chamber not in use
MC - 750 - low ash	113	168	55	32.8	Stage II unit 750°C - low-temperature discharge chamber
WC - 750	72	100	28	28.1	Stage II unit 750°C - low-temperature discharge chamber
SWP I - 550 - VC	45	53	8.2	15.4	Stage III unit - pipe not insulated – backflow of vapours
DNX - 750	41	67	26	39.0	Stage II unit 750°C - low-temperature discharge chamber
DW - 750	30	48	18	37.5	Stage II unit 750°C - low-temperature discharge chamber
MC - 750 - high ash	23	49	26	52.9	Stage II unit 750°C - low-temperature discharge chamber
SWP I - 550 - LC	18	28	9.9	35.5	Stage III unit - discharge chamber not insulated
MC - 750 - wet	16	53	37	70.4	Stage II unit 750°C - low-temperature discharge chamber
WSP II-350-10-0	15	52	37	71.3	?

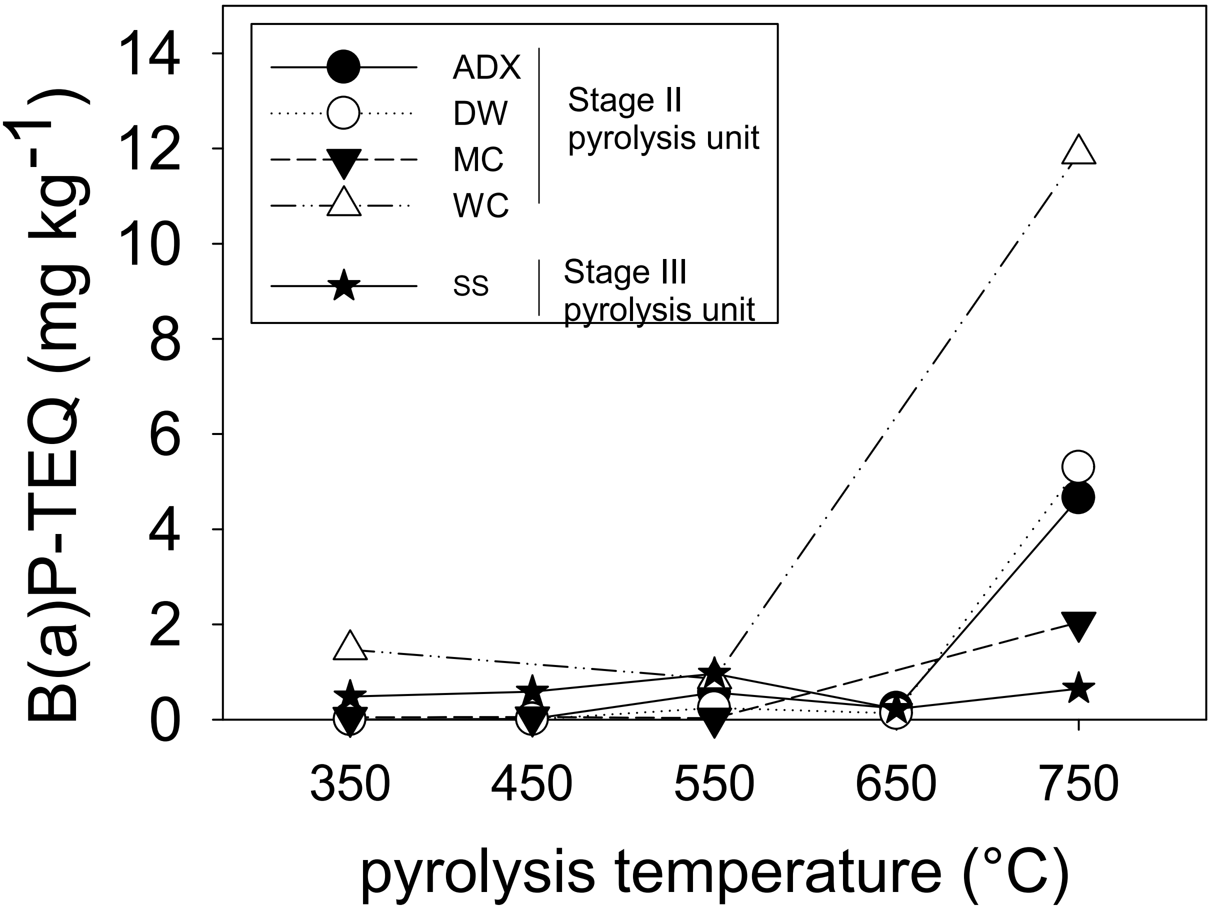

Figure S3: Effect of pyrolysis temperature on TEQ of PAHs (mg kg^{-1}) in biochars from different feedstocks. The biochars were produced from four feedstocks in the Stage II pyrolysis unit (ADX, *Arundo donax*; DW, demolition wood; MC, miscanthus chips; WC, willow chips) and one feedstock in the Stage III pyrolysis unit (SS, sewage sludge).

References

Buss, W., Graham, M.C., MacKinnon, G., Mašek, O., 2016a. Strategies for producing biochars with minimum PAH contamination. J. Anal. Appl. Pyrolysis 119, 24–30. https://doi.org/10.1016/j.jaap.2016.04.001

Buss, W., Graham, M.C., Shepherd, J.G., Mašek, O., 2016b. Suitability of marginal biomass-derived biochars for soil amendment. Sci. Total Environ. 547, 314–322. https://doi.org/doi:10.1016/j.scitotenv.2015.11.148

Buss, W., Mašek, O., 2016. High-VOC biochar – Effectiveness of post-treatment measures and potential health risks related to handling and storage. Environ. Sci. Pollut. Res. 23, 19580–19589. https://doi.org/10.1007/s11356-016-7112-4

Buss, W., Mašek, O., 2014. Mobile organic compounds in biochar – a potential source of contamination – phytotoxic effects on cress seed (Lepidium sativum) germination. J. Environ. Manage. 137, 111–119. https://doi.org/10.1016/j.jenvman.2014.01.045

Buss, W., Mašek, O., Graham, M., Wüst, D., 2015. Inherent organic compounds in biochar–Their content, composition and potential toxic effects. J. Environ. Manage. 156, 150–157. https://doi.org/10.1016/j.jenvman.2015.03.035

Crombie, K., Mašek, O., Sohi, S.P., Brownsort, P., Cross, A., 2013. The effect of pyrolysis conditions on biochar stability as determined by three methods. GCB Bioenergy 5, 122–131. https://doi.org/10.1111/gcbb.12030
